# Ambulatory negative pressure wound therapy of subcutaneous abdominal wounds after surgery: results of the SAWHI randomized clinical trial

**DOI:** 10.1186/s12893-022-01863-x

**Published:** 2022-12-12

**Authors:** Dörthe Seidel, the SAWHI study group, Stephan Diedrich, Florian Herrle, Henryk Thielemann, Frank Marusch, Rebekka Schirren, Recca Talaulicar, Tobias Gehrig, Nadja Lehwald-Tywuschik, Matthias Glanemann, Jörg Bunse, Martin Hüttemann, Chris Braumann, Oleg Heizmann, Marc Miserez, Thomas Krönert, Stephan Gretschel

**Affiliations:** Institut Für Forschung in der Operativen Medizin, Witten/Herdecke University, Cologne, Germany

**Keywords:** Negative pressure wound therapy, Outpatients, Ambulatory care, Abdomen, Wound healing

## Abstract

**Background:**

The SAWHI study showed that negative pressure wound therapy (NPWT) reduced treatment time by 7.8 days and had a 20.2% higher wound closure rate, but required a 2.1-day longer hospital stay than conventional wound treatment (CWT). The majority of study participants began treatment in the hospital and were discharged within 42 days.

**Methods:**

As an add-on to a multicenter randomized clinical trial, selected aspects of hospital discharge, outpatient treatment continuation, and subsequent wound closure outcomes are compared between the treatment arms in patients with subcutaneous abdominal wound healing impairment after surgery without fascia dehiscence in the per protocol population.

**Results:**

Within 42 days, wound closure rates were higher for outpatients in the NPWT arm than for outpatients in the CWT arm (27 of 55 [49.1%]) for both outpatient continuation of NPWT (8 of 26 [30.8%]) and outpatient CWT after NPWT was finished (27 of 121 [22.3%]). Time to wound closure was shorter for outpatients in the NPWT arm (outpatient transfer with: NPWT Mean ± standard error 28.8 ± 8.0 days; CWT 28.9 ± 9.5 days) than in the conventional treatment arm (30.4 ± 8.0 days). Nevertheless, within 30 study sites with patient enrollment, outpatient NPWT was performed in only 20 study sites for 65 of 157 study participants in the treatment arm.

**Conclusions:**

Outpatient NPWT of postsurgical abdominal wounds with healing impairment is feasible and successful and should be encouraged whenever possible. Study site specific avoidance of outpatient NPWT emerges as an additional reason for the prolonged hospitalization time.

*Trial Registration ClinicalTrials.gov Identifier* NCT01528033. Date of registration: February 7, 2012, retrospectively registered

**Supplementary Information:**

The online version contains supplementary material available at 10.1186/s12893-022-01863-x.

## Background

Wound dehiscence with and without infection is a common surgical complication, which frequently occurs after abdominal procedures especially colorectal surgery [1–3]. Depending on the cause of the impaired wound healing, abdominal wound dehiscence may involve only the skin and subcutaneous adipose tissue or may extend to the underlying muscles and fascia [4]. Subcutaneous abdominal wound healing impairment (SAWHI) after surgery requires immediate treatment to prevent further deterioration of the wound and to avoid complications such as fascial dehiscence. Wound complications usually occur from the fourth day after surgery [4]. The diagnosis is often made in the hospital, and wound treatment starts during inpatient care. Since impaired wound healing increases hospitalization time [5, 6] and incurs considerable extra health care costs associated with additional hospital days [7], treatment is continued in outpatient care whenever a patient's health condition permits. Thus, a substantial number of postsurgical abdominal wounds are treated in outpatient care facilities of hospitals and in the home care setting [8–10], but transferring patients to outpatient care still poses challenges for clinicians.

With adequate treatment, wounds heal by secondary intention until full epithelialization or until delayed primary (tertiary) closure. Most patients receive conventional dressings for wound treatment.

Negative pressure wound therapy (NPWT) is an advanced clinical wound care method that has been shown to be a potentially effective alternative to conventional wound treatment (CWT) in many studies in various indications, which have been regularly summarized in systematic reviews [11–16]. A dressing is placed in the wound cavity and the area is sealed with an adhesive sheet [11]. A tube is connected to a negative pressure device that creates a controlled negative pressure in the range of − 50 mmHg to − 125 mmHg. A negative pressure of 125 mmHg has been shown to produce a maximum increase in blood flow [17]. The beneficial effects of NPWT on wound healing have been demonstrated in several basic studies [18, 19]. In practical application, NPWT demonstrates its benefits by promoting granulation tissue formation, reducing the frequency of dressing changes, removing large amounts of wound exudate, and reducing odor [11, 20].

Although NPWT has been increasingly used successfully in the outpatient setting in recent years [21–24], most studies have been conducted in the hospital setting. To date, few randomized clinical trials (RCTs) which included mainly deep peri-vascular groin infections, pressure ulcers, pilonidal sinus wounds, and diabetic foot ulcers have been conducted exclusively in an outpatient setting [25, 26], investigated both hospitalized and ambulatory patients [27–32], or started in hospital and were continued in outpatient care [33–35]. Only two RCTs included nonprimary closable abdominal wounds [20] and surgical abdominal wounds [21, 22] among other wound types. Endpoints comprised mortality [27, 28, 33], wound closure [26–28, 30, 32, 33], adverse events [26–30, 32, 33], amputation [28–30, 32, 33], hospital stay and readmission [30, 32, 33], pain [25–30], physical functioning [25, 26], and health-related quality of life [28, 30]. None of these RCTs provided detailed information on the transfer process to outpatient care or information on length of hospital stay for this specific wound type. Two, nonrandomized clinical trials that included postsurgical abdominal wounds [36, 37] reported hospital length of stay; however, the length of stay specifically attributable to abdominal wounds was not reported or the description of the type of surgical or abdominal wound was not precise.

Until October 1, 2020, NPWT was reimbursable in Germany only in hospitals. During that time, outpatient reimbursement required time-consuming individual applications to the statutory health insurance funds, which were often rejected due to a lack of evidence for the effectiveness of NPWT. In The Netherlands some smaller single-use NPWT-devices were part of the outpatient reimbursement list during the study and in Belgium no outpatient reimbursement of NPWT was available. This insufficient outpatient reimbursement situation resulted in patients who started NPWT in hospital not being discharged from hospital at the earliest possible time, remaining full inpatients with NPWT, or being discharged from hospital only after completing NPWT. In addition, there was a lack of both experience and infrastructure for NPWT transition and outpatient care in some regions of Germany because NPWT in the outpatient sector was not generally reimbursed and therefore not an established treatment option. In particular, the problem of providing enough trained and specialized care services arose, since usage and dressing changes for NPWT requires experience and special skills and in contrast to other wound care products the patient or a relative cannot do the dressing change on their own.

The SAWHI-RCT compared NPWT and CWT in subcutaneous abdominal wounds without fascia dehiscence after surgery with special focus on outcomes and benefits of the treatment options when used across care settings [38]. With NPWT, wound closure time was significantly shorter and wound closure rate was significantly higher [39]. The resource use analysis revealed that the majority of study participants of the PP population who began treatment in the hospital were discharged within 42 days and demonstrated that a large number of outpatient care resources were used [40]. Although the mean treatment length was 7.8 days shorter with NPWT (p < 0.001 U test), the mean hospitalization time within 42 days after randomization was 2.1 days shorter with CWT (p = 0.047 U test). In the CWT arm, 4.2% more participants were discharged from hospital within 42 days, but time to discharge was only 0.9 days longer in the NPWT arm (p = 0.306 U test). More study participants in the NPWT arm (17 (10.8%)) than in the CWT arm (7 (4.0%)) who were treated exclusively in the hospital and more wounds that were surgically closed in the NPWT arm mainly during hospitalization have already been discussed as possible reasons for the prolonged hospital stay.

The aim of this additional analysis of selected data from the SAWHI study was to evaluate the wound closure outcome for study participants who continued their wound treatment in outpatient care and to further investigate the reasons for missing and delayed hospital discharge with NPWT. Because reference data for hospitalization time of patients with SAWHI are lacking in the literature, the reference length of hospital stay was calculated from routine clinical data to get an indication of whether the study data correspond to routine clinical data. Previous and actual results are discussed in the context of the reimbursement situation during the study.

## Methods

### Study design

This additional evaluation was performed using data from the SAWHI randomized clinical trial which was conducted in 34 abdominal surgical departments in Germany, Belgium, and the Netherlands. The study protocol and the informed consent documents were approved by the lead ethical committee of the University of Witten/Herdecke. The trial was registered with the ClinicalTrials.gov Identifier: NCT01528033 on 07.02.2012. The study protocol [38], the effectiveness and safety results [39], and the resource use analysis results [40] were previously published.

### Participants

Information on study participants has been previously published [38–40] and is repeated here for ease of reference: Adult patients (age ≥ 18 years) with spontaneous wound dehiscence, reopened suture, or open wounds that could not be closed by primary intention after abdominal surgery were screened for study participation. Patients with a minimum wound size applicable for both treatment arms were eligible to be included in the study. Inclusion, randomization, debridement or thorough wound cleansing, and initiation of treatment were to be performed within 48 h after diagnosis of SAWHI. Reasons for exclusion were as follows: non-closable defect of the abdominal fascia; expected noncompliance with the protocol and study-related requirements; participation in another trial, which was thought to interfere with the study procedures, patient’s compliance, wound healing, or targeted end points; concomitant therapies or procedures deviating from the standard clinical wound care or with investigational character within 30 days prior to screening; the need for concomitant therapies or procedures directly affecting wound healing; pregnancy, any pre-existing or ongoing organ system failure, that could not be stabilized or solved by appropriate medical treatment; unremovable necrotic tissue present in the study wound; non-enteric or unexplored fistulas; malignancy of the wound and the use of any other NPWT devices on the study wound within 8 days prior to screening.

### Randomization and masking

Information on randomization and masking has been previously published [38–40] and is repeated here for ease of reference: After providing written informed consent, patients were randomly allocated to NPWT or CWT in a 1:1 ratio using a computer-generated list (randomly arranged permuted blocks of variable length) created by the trial statistician and located on a centralized web-based tool hosted by a professional information technology service. Patients were stratified by study site and wound size (≤ 60 cm^3^ and > 60 cm^3^). Each registered investigator received individual access to the tool without knowing the randomization sequence, which ensured allocation concealment. The investigators were responsible for adequately implementing the assigned therapy. Neither study participants, medical staff nor resource use outcome assessors were blinded to the treatment assignment.

### Procedures

After wound debridement or thorough wound cleansing, study treatment started either in-hospital or in an outpatient setting and was to be continued in outpatient care whenever possible.

In the intervention arm, commercially available CE-marked NPWT systems (3 M™ V.A.C.^®^ Therapy, 3 M Company, St. Paul, MN, USA) and consumables, were used at the discretion of the clinical investigator and according to the manufacturer’s instructions. NPWT as interim therapy was discontinued once the condition of a wound was suitable for closing, either by epithelialization or surgically.

The control arm was any CWT regularly used in the respective study site that did not have an experimental status. CWT was applied according to the hospitals’ local clinical standards and guidelines. More details on NPWT and a detailed listing of the dressing materials used in the CWT arm were reported in the resource use publication of the SAWHI study [40].

In both treatment arms, wound-related procedures (wound cleansing; wound lavage; sharp, surgical, autolytic, biological, enzymatic, or mechanical debridement; drainage application) were performed when considered clinically necessary.

All study participants that were eligible for outpatient care and had reasonable access to it, were to be transferred to outpatient care during the study treatment period of 42 days.

Wounds were closed either surgically or by secondary intention. In the NPWT arm, secondary healing was achieved with CWT dressings after NPWT was discontinued.

Baseline assessment was performed during the screening and the randomization visit. Continuous study visits were performed weekly until complete, verified and sustained wound closure or the end of the maximum study treatment time after 42 (+ 3) days. In the event of hospital discharge, end of treatment, wound closure, wound closure confirmation and in case of premature study termination, additional study visits were performed. All study participants were followed for 132 days after randomization.

### Outcomes

#### Inpatient and outpatient care of the study participants per study site

For each study site, the included patients were listed and divided into exclusive inpatients and outpatients. For NPWT, it is also indicated whether the treatment was performed on an outpatient basis.

#### Study participants treated exclusively inpatient

Within each weekly study visit and at end of treatment, the clinical investigators were asked to document whether the study participants were inpatient or outpatient; and if still inpatient, to assess hospital discharge eligibility. In case of being ineligible for discharge, the investigators were asked to document whether continued inpatient treatment was due to the wound condition, the participant`s health condition and comorbidities, or both. Since the study visit at end of maximum treatment was allowed to be performed at day 42 and three subsequent days, information on hospital discharge is partly available until day 45.

#### Reference length of hospital stay in routine care and total length of hospital stay from surgery to end of maximum treatment and observation time of 42 (+ 3) days for the study participants

Reference length of hospital stay from clinical routine data of patients with the same Diagnosis Related Groups (DRGs) were determined. Therefore, based on the German modification of the International Classification of Procedures in Medicine (German: Operationen- und Prozedurenschlüssel = OPS), the underlying main DRGs) were calculated. The OPS codes for each surgical procedure that preceded the wound healing impairment was documented during screening. The categories of surgical procedures to which the respective main OPS codes were assigned are presented as baseline. The OPS 4 digit was used to determine which DRG was most frequently accessed according to the German Institute for the Hospital Remuneration System DRG Browser 2020 (https://www.g-drg.de/). This DRG Browser 2020 is currently based on data derived from participating hospitals in 2018. Only the main OPS codes for the surgical procedures that were declared to be the main intervention were used. To obtain a reference value for the length of hospital stay under routine conditions, an analysis of the DRG data was performed. Average, and lower and upper limits for the maximum reference length of hospital stay were determined. This evaluation was performed for both treatment arms to check whether there is a difference between the treatment arms.

To enable an adequate comparison with the actual hospitalization time of the study participants, an additional calculation was performed that included the hospital days before screening and study inclusion. In the few cases with up to 2 days between screening and randomization, the care status at screening was used for the calculation. In two participants, with outpatient study start, the time between screening and randomization was not added or counted as hospitalization time. If the number of hospitalization days before screening was longer than the time between surgery and screening, hospitalization time was limited to the observation time of interest. Hospitalization days before randomization and during the maximum study treatment time of 42 (+ 3) days were added.

Hospitalization time is reported with mean and standard deviation (SD).

#### Outpatient treatment continuation and subsequent wound closure outcome

Outcomes were reported separately for the treatment options (NPWT in the NPWT arm, CWT in the NPWT arm, NPWT in the CWT arm, and CWT in the CWT arm). Information on complete, verified and sustained wound closures within 42 days as well as overall wound closures within 132 days were provided. Any wound closure documented by the clinical investigator within 132 days that did not conflict with another documented outcome regardless of the criteria defined in the study protocol for complete and verified wound closure lasting at least 14 days was used for analysis. 

Clinical investigators were asked to document the care status of the study participants at the time of final termination of the NPWT period. If the NPWT period was temporarily interrupted, the last day of the last period was used. In caes of an inpatient treatment change, the start time of CWT after NPWT and the time to hospital discharge were provided. 

### Statistical analysis

A total of 539 study participants were randomized to NPWT (273) or CWT (266) in the SAWHI study. Details on the sample size calculation and the planned interim analysis were previously reported in the study protocol and the results of publications [38–40]. The intention to treat (ITT) population included 256 study participants in the NPWT arm and 251 study participants in the CWT arm. The analysis population of choice for this additional evaluation was the per protocol (PP) population, consisting of 331 study participants (NPWT 157; CWT 174) after excluding study participants due to exclusion criteria, unauthorized treatment changes, early treatment termination and incomplete documentation in both treatment arms and NPWT dressing change intervals of more than 72 h in the NPWT arm. Parameters are presented descriptively with mean ± SD or mean (standard error [SE]), median and interquartile range, and minimum and maximum per study participant or per treatment procedure as applicable. Statistical significance was determined as appropriate using the Chi-squared or Mann–Whitney U test respectively with an alpha level of 0.05. SPSS statistical software, version 23 (IBM Inc, Armonk, New York), was used for all analyses.

## Results

Between August 2, 2011, and January 31, 2018, 539 patients were randomized at 34 study sites in Germany, the Netherlands and Belgium. The last follow-up date was June 11, 2018. The 331 study participants (NPWT 157; CWT 174) analyzed in the PP population were enrolled in 30 of the total 34 study sites. Patient flow according to Consolidated Standards of Reporting Trials (CONSORT), including inpatient and outpatient care periods, as well as reasons for screening failure and exclusion from the ITT and PP populations, have been reported in previous results publications [39, 40].

### Demographics and baseline characteristics

Demographic data, major surgical procedures preceding SAWHI categorized by documented OPS codes, and the respective time interval between the surgical procedure and the diagnosis of SAWHI and the start of treatment are provided in Table 1 for the PP population. Most study participants started treatment in hospital. There was no relevant difference between the treatment arms in the frequency of underlying surgical procedures. Intestinal surgery was the most common category with subsequent wound healing impairment.

**Table 1:** Demographics, baseline care status, main surgical procedure that caused the abdominal wound healing impairment and comorbidities at baseline in the PP population

Randomized treatment arms	NPWT	CWT
Study participants in the PP population, No	**157**	**174**
**Age**, median (IQR), years*	64 (19)	66 (18)
**Sex**, No. (%) Male / Female*	96 (61.1) / 61 (38.9)	92 (52.9) / 82 (47.1)
**Main surgical procedure that caused the abdominal wound healing impairment, No. of study participants with OPS codes documented at baseline **(%)	157 (100)	172 (98.9)
OPS categories, No. (%)		
Gastric incision, excision, and resection	4 (2.6)	3 (1.7)
Incision, excision, resection and anastomose of the small and large intestine	58 (36.9)	64 (37.2)
Other operation on the small and large intestine	24 (15.3)	23 (13.4)
Appendix surgery	10 (6.4)	10 (5.8)
Rectal surgery	11 (7.0)	15 (8.7)
Liver surgery	4 (2.6)	5 (2.9)
Gall bladder and bile ducts surgery	14 (8.9)	12 (7.0)
Pancreatic surgery	6 (3.8)	9 (5.2)
Closure of abdominal hernias	11 (7.0)	6 (3.5)
Operations on other abdominal regions	12 (7.6)	18 (10.5)
Incision, excision and closure of blood vessels	0 (0)	1 (0.6)
Application of a shunt and bypass to blood vessels	0 (0)	1 (0.6)
Regional lymphadenectomy (removal of several lymph nodes in a region) as an independent intervention	0 (0)	1 (0.6)
Splenectomy	1 (0.6)	0 (0)
Cystectomy	1 (0.6)	0 (0)
Excision and destruction of prostate tissue	1 (0.6)	0 (0)
Radical prostatovesiculectomy	0 (0)	2 (1.2)
Other incision on skin and subcutaneous tissue	0 (0)	2 (1.2)
**Time between the surgical procedure that preceded the abdominal wound healing impairment and onset of the SAWHI (clinical diagnosis) (days)**, No. Mean (SD) [Min–Max]	156 9.5 (4.7) [0–27]	174 9.8 (7.0) [0 – 74]
**Time between the surgical procedure that preceded the abdominal wound healing impairment and initiation of study treatment (days)**, No. Mean (SD) [Min–Max]	156 10.0 (4.7) [0–28]	174 10.4 (7.1) [0–76]
**Study participants with outpatient study start**, No. (%)^*^	13 (8.3)	19 (10.9)
**Study participants with study start in hospital**, No. (%)^*^	144 (91.7)	155 (89.1)

*Published in: Seidel, D. and R. Lefering (2022). "NPWT Resource Use Compared With Conventional Wound Treatment in Subcutaneous Abdominal Wounds With Healing Impairment After Surgery: SAWHI Randomized Clinical Trial Results." Ann Surg 275(2): e290-e298

### Outpatient treatment continuation and subsequent wound closure outcome in the PP population

In the PP population, 275 of 299 (92.0%) study participants with study start in the hospital were discharged to outpatient care during the study treatment period. A total of 203 of these participants were undergoing treatment at the time of initial hospital discharge (Table 2). The remaining 72 study participants underwent surgical wound closure and/or achieved complete, sustained, and verified wound closure before hospital discharge. One study participant in the CWT arm received CWT on the day of first hospital discharge, but no information on further treatment or care status was available after the discharge day (end of observation at discharge day). Therefore, these study participants are not included in further analysis.

**Table 2:** Treatment continuation and subsequent wound closure outcome after hospital discharge in the PP population

Randomized treatment arm	NPWT		CWT	
Study participants with inpatient study start, No. (%)^*^	144 of 157 (91.7)		155 of 174 (89.1)	
Study participants treated exclusively inpatient, No. (%)^*^	17 of 144 (11.8)		7 of 155 (4.5)	
Study participants with surgical wound closure before first hospital discharge, No. (%)	36 of 144 (25.0)		17 of 155 (11.0)	
**Study participants with hospital discharge**, No. (%)^*^	127 of 144 (88.2)		148 of 155 (95.5)	
**Study participants with completed treatment before first hospital discharge (including those with wound closure)**, No. (%)	46 of 144 (1.9)		26 of 155 (16.8)	
Study participants with completed treatment before first hospital discharge and complete, verified and sustained wound closure within 42 days, No. (%)	40 of 46 (87.0)		21 of 26 (80.8)	
Time to complete, sustained and verified wound closure (days) within 42 days for study participants with completed treatment before first hospital discharge, Mean (SE) [95% CI]	26.3 (1.0) [24.2–28.3]		29.9 (1.5) [26.8–33.0]	
**Study participants under treatment at first hospital discharge**, No. (%)^*^	81 of 144 (56.3)		122 of 155 (78.7)	
Study participants with treatment termination at day of first hospital discharge, No. (%)^*^	0		1	
**Treatment status at first hospital discharge and continuation of the respective treatment during outpatient care in the PP population**	NPWT	CWT after NPWT	NPWT	CWT
Study participants in the outpatient subpopulation, No. (%)	55 of 157 (35.0)	26 of 157 (16.6)	0 of 174 (0)	121 of 174 (69.5)
Total treatment time until complete, sustained and verified wound closure or end of study treatment time after 42 days, Mean (SD) [Min–Max]	25.2 (13.1) [6–42]	34.3 (9.2) [16–42]	NA	34.4 (11.1) [7–42]
Outpatient treatment time until complete, sustained and verified wound closure or end of study treatment time after 42 days, Mean (SD) [Min–Max]	17.1 (11.5) [0–39]	16.1 (9.8) [1–33]	NA	23.1 (11.7) [0–40]
Study participants with rehospitalization, No. (%)	8 of 55 (14.5%)	6 of 26 (23.1%)	NA	13 of 121 (10.7%)
**Study participants with complete, verified and sustained wound closure within 42 days**, No. (%)	27 of 55 (49.1)	8 of 26 (30.8)	NA	27 of 121 (22.3)
Time to complete, sustained and verified wound closure (days) within 42 days, Mean (SE) [95% CI]	28.8 (8.0) [25.6–32.0]	28.9 (9.5) [20.9–36.8]	NA	30.4 (8.0) [27.3–33.6]
**Study participants still under treatment at day 42**, No. (%)	7 of 157 (4.5%)	38 of 157 (24.2%)	NA	85 of 174 (48.9%)
Study participants still under treatment in hospital at day 42, No. (%)	2 of 7 (28.6%)	6 of 38 (15.8%)	NA	8 of 85 (9.4%)
Study participants still under treatment in outpatient care at day 42 No. (%)	5 of 7 (71.4%)	32 of 38 (84.2%)	NA	77 of 85 (90.6%)
**Study participants with wound closure within 132 days**, No. (%)	47 of 55 (85.5)	21 of 26 (80.8)	NA	98 of 121 (81.0)
Time to wound closure (days) within 132 days, Mean (SE) [95% CI]	42.4 (3.3) [35.8–48.9]	54.7 (7.0) [40.2–69.3]	NA	56.3 (2.6) [51.1–61.5]

*Published in: Seidel, D. and R. Lefering (2022). “NPWT Resource Use Compared With Conventional Wound Treatment in Subcutaneous Abdominal Wounds With Healing Impairment After Surgery: SAWHI Randomized Clinical Trial Results.” Ann Surg 275(2): e290-e298

Treatment time was shorter in study participants with outpatient NPWT than in those with outpatient CWT after NPWT and with CWT alone (Table 2). Outpatient treatment time was shorter in the NPWT arm than in the CWT arm for both study participants who transitioned to outpatient care with NPWT and with outpatient CWT after NPWT.

The rehospitalization rates were highest among study participants who were transferred to outpatient care with CWT after completion of NPWT (Table 2).

Wound closure was achieved in approximately 50% of all study participants who continued NPWT in ambulatory care (Table 2). Wound closure rates were higher in the NPWT arm than in the CWT arm for both study participants with outpatient NPWT and with CWT after NPWT in the ambulatory care setting. Time to complete, sustained, and verified wound closure was approximately the same in study participants with outpatient NPWT and those with CWT after NPWT in the outpatient setting. Time to wound closure was shorter for outpatients in the NPWT arm than for outpatients in the CWT arm.

The majority of study participants who were still receiving treatment at the end of the 42-day observation period were in outpatient care (Table 2).

The wound closure rate within 132 days was highest among study participants who continued NPWT in the outpatient setting, whereas the time to wound closure was shortest among these study participants (Table 2).

In the case of healing by secondary intention, NPWT is followed by CWT until complete wound closure. This treatment-final CWT period was mainly performed in ambulatory care settings (Table 3).

**Table 3:** Treatment final CWT period after NPWT in the PP population

Randomized treatment arm	NPWT
Study participants with NPWT in the PP population, No. (%)	157 of 157 (100%)
Study participants with NPWT and interim CWT period, No	1^*^
**Study participants with NPWT and treatment-final CWT period**, No. (%)	63 of 157 (40.1%)
**Start of treatment-final CWT period after NPWT (day)**, No	63
Mean (SD)	16.1 (8.3)
Min–Max	2–34
**Study participants with NPWT and treatment-final CWT period starting during inpatient care**, No. (%)	30 of 63 (47.6%)
**Time between start of the treatment-final CWT period after NPWT and hospital discharge (if applicable) (days)**, No	30
Mean (SD)	9.8 (10.0)
Min–Max	1–41
**Study participants with NPWT and treatment-final CWT period starting during outpatient care**, No. (%)	33 of 63 (52.4%)
**Total length of treatment-final CWT period after NPWT (days)**, No	63
Mean (SD)	20.4 (9.2)
Min–Max	3 – 41
**Length of inpatient treatment-final CWT period after NPWT (days)**, No	63^+^
Mean (SD)	4.4 (6.6)
Min–Max	0–25
**Length of outpatient treatment-final CWT period after NPWT (days)**, No	63^#^
Mean (SD)	16.0 (10.6)
Min–Max	0–39

*One study participant began the study treatment period with 22 days of NPWT, was without treatment for 8 days, then received CWT for 7 days, and finally was treated again with NPWT until the end of the maximum study treatment period

^+^Study participants without inpatient post NPWT CWT period are included with 0 days of treatment

^#^Study participants without outpatient post NPWT CWT period are included with 0 days of treatment

Treatment duration and time to wound closure were shorter in study participants treated exclusively outpatient in the NPWT arm than in participants treated exclusively outpatient in the CWT arm. The wound closure rate within 42 days was higher in participants treated exclusively outpatient in the NPWT arm than in participants treated exclusively outpatient in the CWT arm (Table 4).

**Table 4:** Treatment time and wound closure outcome for study participants treated exclusively in the hospital and in outpatient care in the PP population

Treatment status of the study participants in the PP population	Exclusively treated in hospital in the NPWT arm	Exclusively treated outpatient in the NPWT arm	Exclusively treated in hospital in the CWT arm	Exclusively treated outpatient in the CWT arm
**Treatment time until complete, sustained and verified wound closure or end study treatment time after 42 days (days)**, No. Mean (SD) [Min–Max]	17 21.6 (13.8) [4–42]	13 26.4 (13.2) [7–42]	7 34.9 (12.5) [12–42]	16 33.8 (10.5) [14–42]
**Study participants with complete, verified and sustained wound closure within 42 days**, No. (%)	3 of 17 (17.6)	5 of 13 (38.5)	2 of 7 (28.6)	4 of 16 (25.0)
**Time to complete, sustained and verified wound closure (days) within 42 days (days)**, No. Mean (SE) [95% CI]	3 22.3 (2.7) [10.6–34.1]	5 26.4 (8.9) [15.3–37.5]	2 26.5 (1.5) [7.4–45.6]	4 36.0 (4.0) [29.6–42.4]
Study participants with wound closure within 132 days*, No. (%)	14 of 17 (82.4)	10 of 13 (76.9)	5 of 7 (71.4)	13 of 16 (81.3)
Time to wound closure (days) within 132 days* (days), No. Mean (SE) [95% CI]	14 40.7 (6.4) [26.9–54.4]	10 40.50 (8.0) [22.3–58.7]	5 58.6 (14.1) [19.4–97.8]	13 54.2 (6.4) [40.5–67.9]

*In addition to the clinical effectiveness analysis on complete, verified and for a minimum of 14 days sustained wound closures within 42 days, the number of closed wounds within 132 days was determined. Any wound closure documented by the clinical investigator that did not conflict with another documented outcome was considered

Study participants with outpatient study initiation and outpatient-only treatment in the NPWT arm were identical. In the CWT arm, 19 study participants began treatment as outpatients, of whom 3 participants were hospitalized during treatment. Among these 19 patients with outpatient study start in the CWT arm, treatment time to complete, sustained and verified wound closure at the end study treatment time after 42 days (+ 3 days) averaged (SD) 31.4 (11.3) [min–max 14–42]. Within 42 days, 7 of these 19 participants achieved complete, verified and sustained wound closure within a mean (SE) of 34.0 (1.5) days (95% CI 30.4–37.6). Within 132 days, 6 of these 19 study participants achieved wound closure within a mean (SE) of 50.2 (5.6) days (95% CI 38.2–62.1).

### Study participants treated exclusively inpatient

Among study participants treated exclusively in the hospital, 13 of 17 (76.5%) study participants with NPWT and 5 of 7 (71.4%) study participants with CWT were documented to be ineligible for hospital discharge throughout the treatment period. In 4 of 17 (23.5%) study participants in the NPWT arm and in 2 of 7 (28.6%) study participants with CWT, the status changed from ineligibility to eligible for hospital discharge. In the NPWT arm, health status alone was cited as a reason for not being eligible for hospital discharge in 7 of 17 (41.2%) participants, health status and wound in 6 of 17 (35.3%) participants, and wound alone in 4 of 17 (23.5%) participants. With CWT, for 3 of 7 (42.9%) study participants health only and for 4 of 7 (57.1%) study participants health and wound were reported as the reason for non-eligibility for outpatient care.

After excluding participants treated as inpatient only, length of hospital stay was only 0.9 days shorter with CWT (p = 0.219) (NPWT arm N = 127 mean 11.6 days (SD 9.1 days) min–max [0–40]; CWT arm N = 151 mean 10.7 days (SD 9.5 days) min–max [0–41]).

### Study participants in inpatient and outpatient care per study site in the PP population

Within 30 hospitals with patient enrollment, 157 patients were randomized to NPWT in 27 study sites (Additional file 1: Table S1). In the NPWT arm, 140 of 157 (89.2%) study participants were on outpatient treatment, but only 65 of these 140 outpatients (46.4%) received outpatient NPWT at 20 study sites. 75 study participants continued their treatment as outpatients only after NPWT was completed. In 7 study sites with 36 patients randomized to NPWT, no outpatient NPWT was performed. 2 of these study sites included 10 or more patients. In 13 study sites, 17 of 157 study participants (10.8%) were exclusively inpatients receiving NPWT and in 6 study sites 7 of 174 (4.0%) study participants were exclusively inpatients receiving CWT. There was no clustering of study participants treated exclusively hospitalized at any study site.

### Calculated reference hospitalization time for routine patients with the same surgical procedures and hospitalization time between surgery and end of maximum study treatment time

The total length of hospital stay between surgeries preceding SAWHI and the end of the maximum study treatment period of 42 (+ 3) days was 21.6 (13.0) days (N = 157; min–max 0–62) in the NPWT arm and 19.9 (12.9) days (N = 174; min–max 0–65) in the CWT arm (mean difference 1.7 days; p = 0.117 U test).

The mean reference length of stay for patients registered in the 2018 cost estimate by hospitals with the same major DRGs was 10.8 (5.5) days when OPS codes in the NPWT arm were used and 11.1 (5.0) days when using the OPS codes in the CWT arm (Additional file 1: Table S2). The upper limit of reference length of stay was 20.7 (9.0) days for the NPWT arm OPS codes and 21.5 (8.5) for the CWT arm OPS codes.

## Discussion

Considering the reduced overall treatment time of study participants discharged with NPWT and the subsequent outcome of more frequent and faster wound closure, the benefits of using NPWT in an outpatient setting are evident. The results of previous noncontrolled studies with other wound types, which demonstrated that outpatient NPWT is feasible and successful, were confirmed by the results of the additional analysis of the outpatient data from the SAWHI study [21, 24, 37]. Outpatient NPWT was superior in terms of treatment time and wound closure outcome, both within the study treatment period of 42 days and within the total observation period of 132 days, even for study participants treated exclusively as outpatients. The analysis of wound closure within the overall observation period of 132 days was performed although the documentation is limited to a simple date documented by the clinical investigators, which limits the validity of the results.

Nevertheless, hospitalization time was longer in the NPWT arm than in the CWT arm [39, 40]. Hospital discharge was slightly delayed with NPWT. The results of a 2009 retrospective chart review by de Leon comparing NPWT and advanced moist wound care for postoperative wounds also concluded that NPWT had a shorter wound healing time, but hospital length of stay was longer [36]. However, the cause of this effect was not discussed in de Leon's study. In the SAWHI study, significantly more study participants with CWT were treated in outpatient care. More study participants with NPWT were treated exclusively inpatient than with CWT. More wounds were closed surgically mainly during hospitalization in the NPWT arm.

In addition to these previously identified reasons for the prolonged hospitalization time with NPWT, additional analysis of selected data from the SAWHI study revealed a study-site-specific avoidance of outpatient NPWT and possible selection of study participants for outpatient transfer. While most study participants were transferred to outpatient care during the study, only 41.4% received outpatient NPWT in 20 of 30 study sites with patient enrollment. Some study sites transferred participants to outpatient care only after final discontinuation of NPWT.

For the majority of inpatient-only study participants, health status was the reason for continued hospitalization, but 23.5% of inpatient-only participants with NPWT were kept in the hospital solely because of the wound, whereas the wound was not the sole reason for lack of hospital discharge in any of the inpatient-only study participants in the CWT arm.

The analysis of hospitalization time was repeated with a different starting point to allow comparison with routine data. The mean hospital length of stay between randomization and the end of the maximum treatment period was 2.1 days shorter with CWT than with NPWT [40], and even when the observation period was extended by including the preceding surgical procedure, a difference in hospital length of stay of 1.7 days remained between the treatment arms but was no longer significant. Compared with the average reference hospital length of stay of routine patients, the actual mean hospitalization time after the surgery preceding the SAWHI was 10.8 days longer in the NPWT arm and 8.8 days longer in the CWT arm. The overall prolonged hospital stay reflects the fact that only participants with wound healing impairment were included in the study, whereas under routine conditions only a part of the population develops a wound complication after surgery. Only the main OPS codes were used to identify the relevant DRGs. Secondary diagnoses, and other procedures could change the severity of the cases, resulting in different DRGs with different lengths of stay. However, as only an orienting reference range was to be created for the estimation of the duration of hospitalization present in the study, the values determined with the described method were considered sufficiently precise. Noting that 45 study participants with NPWT (28.7%) and 85 study participants with CWT (48.9%) were still under treatment after Day 42, the actual hospital length of stay is most likely higher than what our analysis reports. Unfortunately, this shows once again that the 42-day treatment and observation time was chosen too short. Despite this, hospitalization times in both treatment arms are in the upper limit range of the reference length of stay for patients registered in the 2018 calculation by hospitals with the same main DRGs.

The problems with outpatient transfers caused by the lack of outpatient reimbursement obviously exist both in the study and in routine care and could unfortunately only be influenced to a small extent by the countermeasures applied in the study in the form of support by outpatient care services, assistance with applications at the statutory health insurance funds, and reimbursement of medical devices and consumables in case of rejection of these applications. Furthermore, the time needed to adapt the hospital’s internal procedures and reactivate or establish outpatient cooperation was probably too short to ensure the transition of all eligible participants with NPWT to outpatient care from the beginning. However, the existing opinion that patients can be discharged earlier to outpatient care with NPWT is supported by the results of the present analysis [22, 23].

Following a decision by the Federal Joint Committee (G-BA) in 2020 (https://www.g-ba.de/beschluesse/4085/), NPWT is now available as a service of certain office-based specialists in diabetology, vascular surgery, and dermatology and outpatient reimbursement of NPWT is part of the benefits catalog of the statutory health insurance funds in Germany since October 1, 2020. This is a benefit to patients and relieves health care providers of case-by-case requests. However, it is necessary to improve the outpatient infrastructure in the coming years to adequately implement the application of this new freedom. Lack of outpatient organizational structures is a possible cause of missing hospital discharge. Considering the advantages of outpatient NPWT in terms of shorter treatment and wound closure time, these barriers should be removed as soon as possible. In Germany, however, the fact that reimbursement for outpatient NPWT is only possible on the order of a physician continues to be a potential barrier to widespread availability of this innovative treatment concept for patients with acute and chronic wounds.

## Conclusions

In this study, outpatient NPWT of postsurgical abdominal wounds with healing impairment was feasible and successful. Study site specific avoidance of outpatient NPWT emerges as an additional reason for the prolonged hospitalization time. NPWT should be continued in the outpatient setting whenever patient’s health permits. However, the process of transferring study patients from hospital to outpatient care was not optimal, especially in the NPWT arm, resulting in longer hospital stays. The previous reimbursement situation and a lack of infrastructure for outpatient NPWT may have biased the results for hospital length of stay and outpatient length of treatment. However, with NPWT performed entirely or partially in an outpatient setting, treatment times were shorter and wound healing outcomes were better than with CWT.

Future studies should evaluate the performance and outcome of outpatient NPWT after the changed reimbursement situation has led to an adjustment of processes in German hospitals. Comparison of these German national data with experiences in other countries, taking into account differences in reimbursement systems and availability of therapies, could provide further valuable insights. In these studies, the type of outpatient care setting in which study participants were followed up after hospital discharge should be recorded to provide reliable and fully comprehensive information on whether study participants were treated in home care, in a rehabilitation facility, by an office-based physician, a nursing service, or in a hospital outpatient facility.

## Supplementary Information

**Additional file 1.** **Table S1:** Study participants with inpatient and outpatient care per study site in the PP population. **Table S2:** Average, and lower and upper limit for the maximum hospitalization time in clinical routine based on the DRGs generated from the main OPS codes of the study participants.

## Data Availability

The datasets used and/or analyzed during the current study are available from the corresponding author on reasonable request.

## Abbreviations

NPWT: Negative pressure wound therapy
CWT: Conventional wound treatment
SAWHI: Subcutaneous abdominal wound healing impairment
RCT: Randomized clinical trial
DRG: Diagnosis related groups
OPS: German: Operationen- und Prozedurenschlüssel
SD: Standard deviation
ITT: Intention to treat
PP: Per protocol
SE: Standard error
CONSORT: Consolidated Standards of Reporting Trials

## References

[1] Sandy-Hodgetts K, Carville K, Leslie GD (2015). Determining risk factors for surgical wound dehiscence: a literature review. Int Wound J.

[2] European Centre for Disease Prevention and Control, *Annual Epidemiological Report 2016—Surgical site infections*. 2016, ECDC: Stockholm.

[3] Smith H (2017). Health care-associated infections studies project: an American Journal of Infection Control and National Healthcare Safety Network data quality collaboration. Am J Infect Control.

[4] World Union of Wound Healing Societies (WUWHS), *Consensus Document. Surgical wound dehiscence: improving prevention and outcomes.* 2018, Wounds International: London.

[5] Coello R (2005). Adverse impact of surgical site infections in English hospitals. J Hosp Infect.

[6] Weber WP (2008). Economic burden of surgical site infections at a European university hospital. Infect Control Hosp Epidemiol.

[7] Jenks PJ (2014). Clinical and economic burden of surgical site infection (SSI) and predicted financial consequences of elimination of SSI from an English hospital. J Hosp Infect.

[8] Chetter IC (2017). A survey of patients with surgical wounds healing by secondary intention; an assessment of prevalence, aetiology, duration and management. J Tissue Viability.

[9] Probst S (2014). EWMA document: home care-wound care: overview, challenges and perspectives. J Wound Care.

[10] Apelqvist J (2017). EWMA document: negative pressure wound therapy. J Wound Care.

[11] Ubbink DT (2008). A systematic review of topical negative pressure therapy for acute and chronic wounds. Br J Surg.

[12] Dumville JC, et al. Negative pressure wound therapy for treating surgical wounds healing by secondary intention. Cochrane Database Syst Rev, 2015(6): CD011278. 10.1002/14651858.CD011278.pub2 PMC10960640 26042534

[13] Webster J (2019). Negative pressure wound therapy for surgical wounds healing by primary closure. Cochrane Database Syst Rev.

[14] Zens Y (2020). Negative pressure wound therapy in patients with wounds healing by secondary intention: a systematic review and meta-analysis of randomised controlled trials. Syst Rev.

[15] Janssen AH (2016). Negative pressure wound therapy versus standard wound care on quality of life: a systematic review. J Wound Care.

[16] Webster J, et al. Negative pressure wound therapy for skin grafts and surgical wounds healing by primary intention. Cochrane Database Syst Rev, 2014(10): CD009261. 10.1002/14651858.CD009261.pub3 25287701

[17] Morykwas MJ (2001). Effects of varying levels of subatmospheric pressure on the rate of granulation tissue formation in experimental wounds in swine. Ann Plast Surg.

[18] Morykwas MJ (1997). Vacuum-assisted closure: a new method for wound control and treatment: animal studies and basic foundation. Ann Plast Surg.

[19] Morykwas MJ (2006). Vacuum-assisted closure: state of basic research and physiologic foundation. Plast Reconstr Surg.

[20] Argenta LC, Morykwas MJ (1997). Vacuum-assisted closure: a new method for wound control and treatment: clinical experience. Ann Plast Surg.

[21] Stryja J (2015). Cost-effectiveness of negative pressure wound therapy in outpatient setting. Rozhl Chir.

[22] Dowsett C (2012). The economic benefits of negative pressure wound therapy in community-based wound care in the NHS. Int Wound J.

[23] Ousey K, Milne J (2009). Negative pressure wound therapy in the community: the debate. Br J Community Nurs.

[24] Tamir E (2018). Outpatient negative-pressure wound therapy following surgical debridement: results and complications. Adv Skin Wound Care.

[25] Banasiewicz T (2013). Portable VAC therapy improve the results of the treatment of the pilonidal sinus–randomized prospective study. Pol Przegl Chir.

[26] Biter LU (2014). The use of negative-pressure wound therapy in pilonidal sinus disease: a randomized controlled trial comparing negative-pressure wound therapy versus standard open wound care after surgical excision. Dis Colon Rectum.

[27] Ashby RL (2012). A pilot randomised controlled trial of negative pressure wound therapy to treat grade III/IV pressure ulcers [ISRCTN69032034]. Trials.

[28] Seidel D (2020). Negative pressure wound therapy compared with standard moist wound care on diabetic foot ulcers in real-life clinical practice: results of the German DiaFu-RCT. BMJ Open.

[29] Mody GN (2008). A blinded, prospective, randomized controlled trial of topical negative pressure wound closure in India. Ostomy Wound Manage.

[30] Arundel C (2018). Pilot feasibility randomized clinical trial of negative-pressure wound therapy versus usual care in patients with surgical wounds healing by secondary intention. BJS Open.

[31] Arundel C (2016). Negative pressure wound therapy versus usual care for Surgical Wounds Healing by Secondary Intention (SWHSI trial): study protocol for a randomised controlled pilot trial. Trials.

[32] Seidel D, Lefering R, g. DiaFu study (2022). NPWT resource use compared with standard moist wound care in diabetic foot wounds: DiaFu randomized clinical trial results. J Foot Ankle Res.

[33] Acosta S, Monsen C, Dencker M (2013). Clinical outcome and microvascular blood flow in VAC(R)—and Sorbalgon(R) -treated peri-vascular infected wounds in the groin after vascular surgery—an early interim analysis. Int Wound J.

[34] Monsen C (2015). A randomised study of NPWT closure versus alginate dressings in peri-vascular groin infections: quality of life, pain and cost. J Wound Care.

[35] Monsen C (2014). Vacuum-assisted wound closure versus alginate for the treatment of deep perivascular wound infections in the groin after vascular surgery. J Vasc Surg.

[36] de Leon JM (2009). Cost-effectiveness of negative pressure wound therapy for postsurgical patients in long-term acute care. Adv Skin Wound Care.

[37] Trueman P (2008). The feasibility of using V.A.C. Therapy in home care patients with surgical and traumatic wounds in the Netherlands. Int Wound J.

[38] Seidel D, Lefering R, Neugebauer EA (2013). Treatment of subcutaneous abdominal wound healing impairment after surgery without fascial dehiscence by vacuum assisted closure (SAWHI-V.A.C.(R)-study) versus standard conventional wound therapy: study protocol for a randomized controlled trial. Trials.

[39] Seidel D (2020). Negative pressure wound therapy vs conventional wound treatment in subcutaneous abdominal wound healing impairment: the SAWHI randomized clinical trial. JAMA Surg.

[40] Seidel D, Lefering R (2022). NPWT resource use compared with conventional wound treatment in subcutaneous abdominal wounds with healing impairment after surgery: SAWHI randomized clinical trial results. Ann Surg.

