# Supplementary material for: Ambulatory negative pressure wound therapy of subcutaneous abdominal wounds after surgery: results of the SAWHI randomized clinical trial
Source: BMC Surg. 2022 Dec 12;22:425. doi: 10.1186/s12893-022-01863-x (PMC9743503; doi:10.1186/s12893-022-01863-x)
Supplement: Supplementary file 1 — Additional file 1. Table S1: Study participants with inpatient and outpatient care per study site in the PP population. Table S2: Average, and lower and upper limit for the maximum hospitalization time in clinical routine based on the DRGs generated from the main OPS codes of the study participants. [file 12893_2022_1863_MOESM1_ESM.docx]

**Supplementary File**

Table S1: Study participants with inpatient and outpatient care per study site in the PP population

| **Randomized treatment** | **NPWT** | | | **CWT** | |
| --- | --- | --- | --- | --- | --- |
| **Study participants in the treatment sector per study site** | **Exclusive inpatients** | **Outpatients*** | **Outpatient NPWT** | **Exclusive inpatients** | **Outpatients*** |
| Study site 1 | 0 | 3 | 0 | 1 | 7 |
| Study site 2 | 0 | 2 | 2 | 0 | 1 |
| Study site 3 | 0 | 7 | 6 | 0 | 11 |
| Study site 5 | 0 | 0 | 0 | 0 | 3 |
| Study site 7 | 2 | 13 | 0 | 0 | 11 |
| Study site 9 | 1 | 9 | 4 | 0 | 14 |
| Study site 10 | 1 | 15 | 7 | 2 | 17 |
| Study site 11 | 1 | 1 | 1 | 1 | 0 |
| Study site 12 | 1 | 12 | 8 | 0 | 11 |
| Study site 13 | 1 | 13 | 6 | 0 | 12 |
| Study site 14 | 1 | 2 | 1 | 0 | 2 |
| Study site 16 | 2 | 7 | 5 | 0 | 4 |
| Study site 18 | 0 | 1 | 1 | 0 | 3 |
| Study site 20 | 0 | 1 | 0 | 0 | 2 |
| Study site 21 | 0 | 1 | 1 | 0 | 0 |
| Study site 22 | 1 | 1 | 0 | 0 | 3 |
| Study site 23 | 2 | 8 | 0 | 0 | 9 |
| Study site 26 | 1 | 0 | 0 | 0 | 0 |
| Study site 27 | 0 | 10 | 6 | 1 | 12 |
| Study site 28 | 0 | 3 | 1 | 0 | 6 |
| Study site 34 | 0 | 4 | 0 | 0 | 1 |
| Study site 35 | 2 | 4 | 3 | 1 | 9 |
| Study site 37 | 0 | 1 | 1 | 0 | 2 |
| Study site 38 | 0 | 6 | 3 | 0 | 7 |
| Study site 39 | 0 | 1 | 1 | 0 | 2 |
| Study site 40 | 0 | 4 | 2 | 0 | 4 |
| Study site 45 | 0 | 0 | 0 | 0 | 1 |
| Study site 46 | 1 | 6 | 6 | 1 | 4 |
| Study site 47 | 0 | 5 | 5 | 0 | 7 |
| Study site 48 | 0 | 0 | 0 | 0 | 2 |
| **Sum** | **17 of 157 (10.8%)** | **140 of 157 (89.2%)** | **65 of 157 (41.4%)** | **7 of 174 (4.0%)** | **167 of 174 (96.0%)** |

*The number of outpatients per study site includes study participants with outpatient study start and those with inpatient study start and hospital discharge during the study treatment of 42 days.

**Table S2: Average, and lower and upper limit for the maximum hospitalization time in clinical routine based on the DRGs generated from the main OPS codes of the study participants**

| **Randomized treatment arms** | **NPWT** | **CWT** |
| --- | --- | --- |
| **Study participants in the PP population, No.** | 157 | 174 |
| **Available DRGs^*^, No.** | 151 | 165 |
| **Lower limit for maximum hospitalization time** |  |  |
| Mean (SD) | 2.9 (1.7) | 3.0 (1.5) |
| Median (IQR) | 3.0 (1.0) | 3.0 (2.0) |
| Min - Max | 1.0 - 12.0 | 1.0 - 12.0 |
| **Average for maximum hospitalization time** |  |  |
| Mean (SD) | 10.8 (5.5) | 11.1 (5.0) |
| Median (IQR) | 12.0 (4.2) | 12.0 (5.6) |
| Min - Max | 2.7 - 39.2 | 2.7 - 39.2 |
| **Upper limit for maximum hospitalization time** |  |  |
| Mean (SD) | 20.7 (9.0) | 21.5 (8.5) |
| Median (IQR) | 23.0 (8.0) | 23.0 (10.0) |
| Min - Max | 6.0 - 57.0 | 6.0 - 57.0 |

* For some of the German procedure classification (German: Operationen- und Prozedurenschlüssel – OPS) codes, no Diagnosis Related Group (DRG) was available due to a split of the codes in the meantime.
